# Chikungunya as a Cause of Acute Febrile Illness in Southern Sri Lanka

**DOI:** 10.1371/journal.pone.0082259

**Published:** 2013-12-02

**Authors:** Megan E. Reller, Ufuoma Akoroda, Ajith Nagahawatte, Vasantha Devasiri, Wasantha Kodikaarachchi, John J. Strouse, Robert Chua, Yan'an Hou, Angelia Chow, October M. Sessions, Truls Østbye, Duane J. Gubler, Christopher W. Woods, Champica Bodinayake

**Affiliations:** 1 Division of Medical Microbiology, Department of Pathology, Johns Hopkins University School of Medicine, Baltimore, Maryland, United States of America; 2 Division of Pediatric Hematology, Department of Pediatrics, Johns Hopkins University School of Medicine, Baltimore, Maryland, United States of America; 3 Medical Faculty of University of Ruhuna, Galle, Sri Lanka; 4 Duke-National University of Singapore Graduate Medical School Program in Emerging Infections, Singapore, Singapore; 5 Department of Community and Family Medicine, Duke University School of Medicine, Durham, North Carolina, United States of America; 6 Division of Infectious Diseases and International Health, Department of Medicine, Duke University School of Medicine, Durham, North Carolina, United States of America; 7 Duke University School of Medicine, Durham, North Carolina, United States of America; 8 Hubert-Yeargan Center for Global Health, Durham, North Carolina, United States of America; University of California Davis, United States of America

## Abstract

**Background:**

Chikungunya virus (CHIKV) re-emerged in Sri Lanka in late 2006 after a 40-year hiatus. We sought to identify and characterize acute chikungunya infection (CHIK) in patients presenting with acute undifferentiated febrile illness in unstudied rural and semi-urban southern Sri Lanka in 2007.

**Methodology/Principal Findings:**

We enrolled febrile patients ≥ 2 years of age, collected uniform epidemiologic and clinical data, and obtained serum samples for serology, virus isolation, and real-time reverse-transcriptase PCR (RT-PCR). Serology on paired acute and convalescent samples identified acute chikungunya infection in 3.5% (28/797) patients without acute dengue virus (DENV) infection, 64.3% (18/28) of which were confirmed by viral isolation and/or real-time RT-PCR. No CHIKV/DENV co-infections were detected among 54 patients with confirmed acute DENV. Sequencing of the E1 coding region of six temporally distinct CHIKV isolates (April through October 2007) showed that all isolates posessed the E1-226A residue and were most closely related to Sri Lankan and Indian isolates from the same time period. Except for more frequent and persistent musculoskeletal symptoms, acute chikungunya infections mimicked DENV and other acute febrile illnesses. Only 12/797 (1.5%) patients had serological evidence of past chikungunya infection.

**Conclusions/Significance:**

Our findings suggest CHIKV is a prominent cause of non-specific acute febrile illness in southern Sri Lanka.

## Introduction

Chikungunya is caused by infection with chikungunya virus (CHIKV), a mosquito-transmitted *Alphavirus*, family *Togaviridae*, found in tropical and subtropical regions of Africa, the Indian Ocean islands, and south and southeast Asia [1]. First recognized in Tanzania in 1953, chikungunya, which translates as “to be contorted” or “that which bends up,” is characterized by fever and musculoskeletal pain, and hence mimics dengue fever and other acute febrile illnesses. In Africa the virus is enzootic and is thought to be maintained in a sylvatic cyle involving primates and forest *Aedes* species [1]. In Asia CHIKV largely causes urban epidemics and circulates between humans and mosquitos (primarily *Aedes aegypti*, but also *Ae. albopictus*); recent evidence suggests it may also have a sylvatic cycle in Malaysia [2]. 

Epidemic CHIKV was recognized again in the Indian subcontinent in 2005, first in India and then in neighboring Sri Lanka (population ~ 20 million) in November 2006 [3]. After a 40-year hiatus [4], >37,000 suspected cases of CHIKV were reported in densely-populated regions in the north, east, and western coastal belt of Sri Lanka in 2007 and a similar number in 2008 [5,6]. Intrinsic limitations in diagnosis, limited resources for surveillance, and a concurrent dengue virus (DENV) epidemic delayed recognition and characterization of the CHIKV outbreak. In a community-based study in the Central Province during this period, in which patients with strongly suspected CHIKV or DENV were enrolled, Kularatne and colleagues found arthritis to be indicative of chikungunya (present in 57% [12/23] with chikungunya versus no one [0/21] with dengue); further, all with arthritis developed chronic sequelae [5].

To investigate and characterize CHIKV as a cause of acute febrile illness in unstudied rural and semi-urban southern Sri Lanka and to compare it with dengue, we prospectively enrolled patients ≥2 years of age with undifferentiated fever who presented to a large public hospital. We obtained epidemiological and clinical data and samples for paired serology and real-time reverse-transcriptase PCR (RT-PCR).

## Methods

### Setting and patients

We recruited patients in the emergency department, acute care clinics, and adult and pediatric wards of Teaching Hospital Karapitiya (THK), southern Sri Lanka’s largest (1300-bed) hospital, located in the seaport city of Galle. Febrile (>38°C tympanic) patients >2 years of age without trauma or hospitalization within the previous 7 days were eligible for enrollment. Study doctors verified eligibility and willingness to return for a 2-4 week convalescent follow-up visit and obtained written consent from patients (>18 years) or parents (<18 years) and assent if ≥12 -17 years. Study personnel recorded epidemiologic and clinical data obtained at enrollment on a standardized form. Study doctors obtained blood for on-site clinician-requested testing and off-site diagnostic testing. Patients returned for clinical and serologic follow-up 2 to 4 weeks later, or if unable to return and address was known, were visited in their home. 

### Samples

Serum samples were stored promptly at -80°C. Samples were shipped on dry ice to the University of North Carolina at Chapel Hill School of Medicine to identify acute DENV infections [7] and then to the Duke-NUS Graduate Medical School Singapore, Program in Emerging Infectious Diseases to diagnose acute CHIKV infections. 

### Testing for CHIKV

Serum samples were tested for CHIKV IgG by initially screening convalescent sera at a dilution of 1:32 by indirect immunofluenscence (IFA); those positive were rescreened at 1:64. If positive at 1:64, acute and convalescent sera were tested concurrently and serially diluted in phosplate-buffered saline to titer to endpoint. Acute-phase serum from patients with acute CHIKV were processed for virus isolation and RT-PCR.

### IgG IFA for CHIKV

CHIKV stock (Ross strain) was grown in baby hamster kidney cells (BHK-21, ATCC CCL-10), harvested, and used to spot 12-well Teflon coated slides. After drying, slides were fixed in cold acetone for 10 minutes, air dried, and stored at -20°C. Diluted patient sera along with negative and positive controls were added (20 ul) to each slide well, incubated at 37°C for 45 minutes, rinsed once with 1X PBS, immersed in 1 xPBS for 5 minutes, and air dried. Twenty µl of FITC-conjugated anti-human IgG antibody with 0.1% Evans Blue were added to each well, incubated at 37°C for 45 min, rinsed in PBS, and a cover glass mounted. The slides were examined using a fluorescent microscope. 

### Serologic interpretation

We considered an IgG titer of 64 as definitive evidence of CHIKV infection. We defined acute CHIKV infection as a 4-fold or greater rise in IgG titer, including in those with seroconversion (i.e. acute-sample titer 16 and convalescent sample-titer ≥ 64). We defined past CHIKV infection as stable (i.e. no change), <4-fold rise, or decreasing IgG titers. 

### Real-time reverse-transcriptase PCR for CHIKV

RNA was extracted from acute-phase serum of patients with serologically confirmed acute CHIKV infection using the QIAamp Viral RNA Mini kit (QIAGEN, Hilden, Germany) according to manufacturer’s instructions.  Reverse transcription was performed using Invitrogen Superscript III First Strand Synthesis System (Life Technologies, Carlsbad, CA), according to manufacturer’s instructions.  Real time-RT-PCR using the LightCycler 480 SYBR Green I Master kit (Roche Diagnostics, Penzberg, Germany) and primers described elsewhere [8] was performed under the following conditions: 95°C for 10 seconds, 55°C for 5 seconds, and 72°C for 10 seconds for 45 cycles before melt curve analysis.  If a clear melting peak for CHIKV (between 85.1°C and 85.7°C) was not observed, the sample was subjected to electrophoresis on a 1.5% agarose gel. A positive control derived from cultured CHIKV was included in the run. A sample displaying an amplification profile within 40 cycles of amplification and bearing similarity to the peak profile of the positive control was considered as positive for CHIKV. Negative controls were included for the reverse transcriptase and real time-RT-PCR steps.  

### Isolation of CHIKV

Virus isolation was done by in-vitro inoculation of C6/36 *Ae. albopictus* cells adapted to a higher temperature to increase virus replication and reduce the incubation period[9]. Acute serum from all confirmed acute cases were diluted 1:10 in L-15 (Gibco, Life Technologies^TM^, Carlsbad, CA, USA) maintenance medium and 200 uL was inoculated onto a confluent monolayer of C6/36 cells. After adsorption for 1 hour at 37°C, the inoculum was removed and fresh medium added. The cells were then incubated at 30°C until cytopathic effects were observed or up to 10 days, whichever was earlier. Viruses were identified by using the culture supernatant for RNA extraction followed by cDNA synthesis and RT-PCR using primers specific for CHIKV.

### Sequencing of the E1 coding region of CHIKV

CHIKV RNA extracted from the virus isolation culture supernatants of 6 patients (2 each enrolled early, mid, and late during the study) was sequenced. The culture supernatant was collected and centrifuged at 2000 rpm for 10 minutes to remove cell debris. Viral RNA was extracted from the supernatant with a QIAamp Viral RNA Mini Kit (QIAGEN, Valencia, CA, USA) according to the manufacturer’s protocol and stored at -80°C until use. Reverse transcription with random hexamers to yield cDNA was performed with Invitrogen Superscript III First Strand Synthesis System (Life Technologies, Carlsbad, CA), according to manufacturer's instructions. CHIKV structural genes were amplified with the following primer pairs: CHIKV-34F and CHIKV-39R, CHIKV-36F and CHIKV-41R, CHIKV-40F and CHIKV-45R, CHIKV-44F and CHIKV-49R, CHIKV-46F and CHIKV-49R. All primer sequences are listed in Table S1. CHIKV RT-PCR products were excised from a 1% preparative agarose gel and extracted with the Qiagen Qiaquick Gel Extraction Kit. RT-PCR products were sequenced with the same forward and reverse primers used for amplification. The resulting sequences are deposited in GenBank with the accession numbers KF578457-KF578462. Consensus sequences were then compared to the published E1 sequences [10,11]. Multiple sequence alignment of the Sri Lankan CHIKV E1 sequences to other CHIKV sequences deposited in GenBank was carried out using a fast Fourier transform in MAFFT [12]. The maximum-likelihood phylogenetic tree was inferred from the sequence alignment using RAxML [13,14]. The robustness of the maximum-likelihood tree was assessed by 1000 maximum-likelihood bootstrap replications. The maximum-likelihood tree was visualized and produced using FigTree v1.4.0 [15]. 

### Statistical Analysis

Proportions were compared using the chi-square test or Fisher’s exact test and continuous variables using ANOVA if normally distributed and Kruskal-Wallis rank sum test if not normally distributed. Analyses were done with Stata IC 11.0 (StataCorp, College Station, TX).

### Ethics Statement

Written consent, and assent when appropriate, was obtained from all participants. The institutional review boards of Ruhuna University (Sri Lanka), Johns Hopkins University, and Duke University Medical Center approved the study.

## Results

### Febrile cohort

Paired sera to identify acute CHIKV infections in those without acute DENVwere available from 797/1079 (73.9%) patients consecutively enrolled between February and November 2007. Those for whom paired sera were available did not differ by age (p=0.33), sex (p=0.10), or level of education (p=0.09) from others. Most participants (90.2%) reported rural residence, a greater proportion of whom returned for follow-up than urban dwellers (75.0% vs. 62.9%, p=0.007). 

Among these 797 patients, most were male (60.7%), and the median age (30.8 years [intraquartile range, IQR 19, 48]) did not differ by sex (p=0.89). Many (35.6%) febrile patients reported having taken an antibiotic for their illness before presentation. The reported duration of fever and of illness before presentation was similar (p=0.66 and p=0.34, respectively) in those with and without follow-up. The median time between the acute visit and convalescent follow-up was 21 days (IQR 15, 33). 

### Acute CHIKV infection

**Figure 1:**
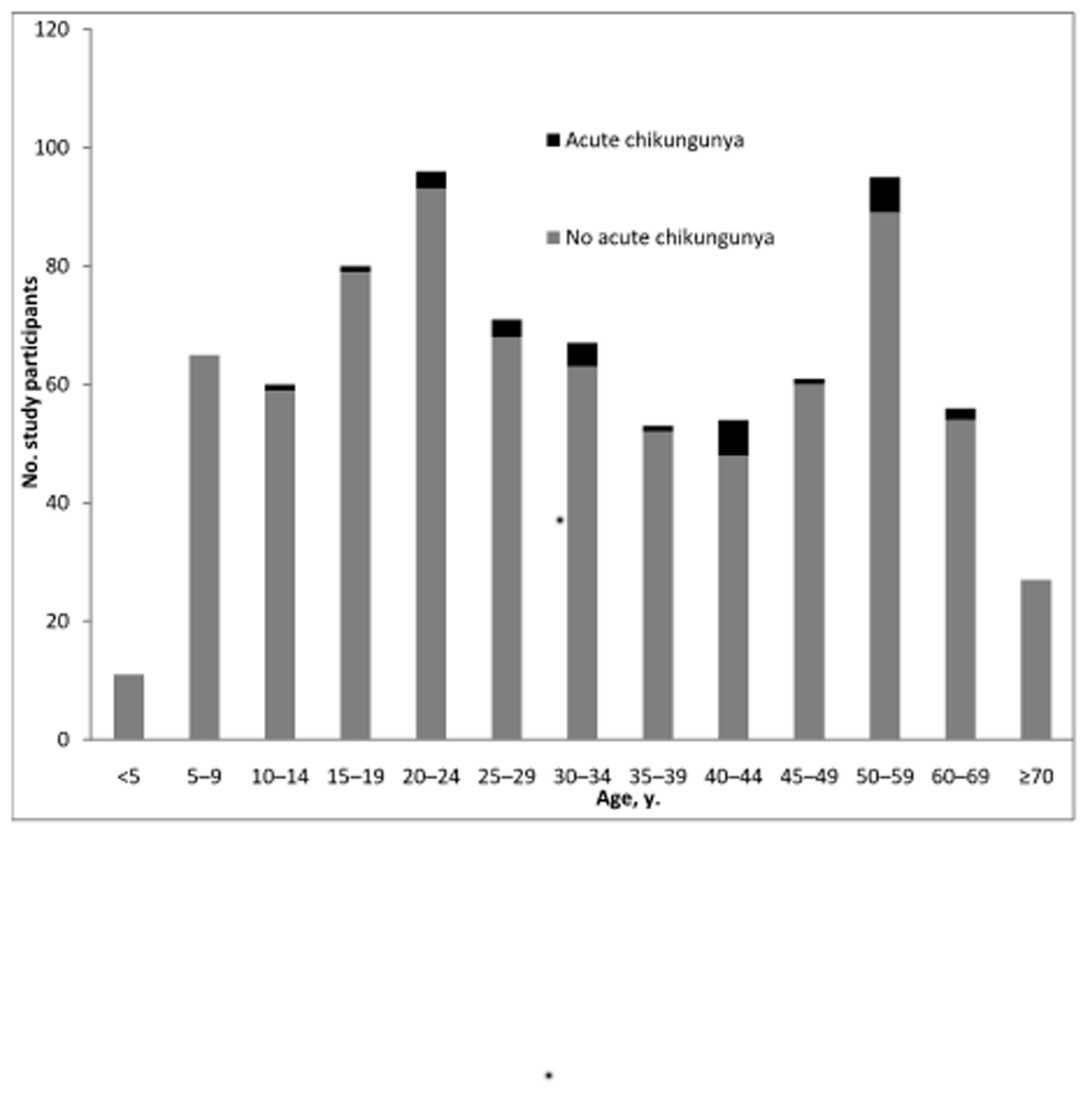
Age distribution of patients with and without acute chikungunya infection, southern Sri Lanka, 2007.

**Figure 2:**
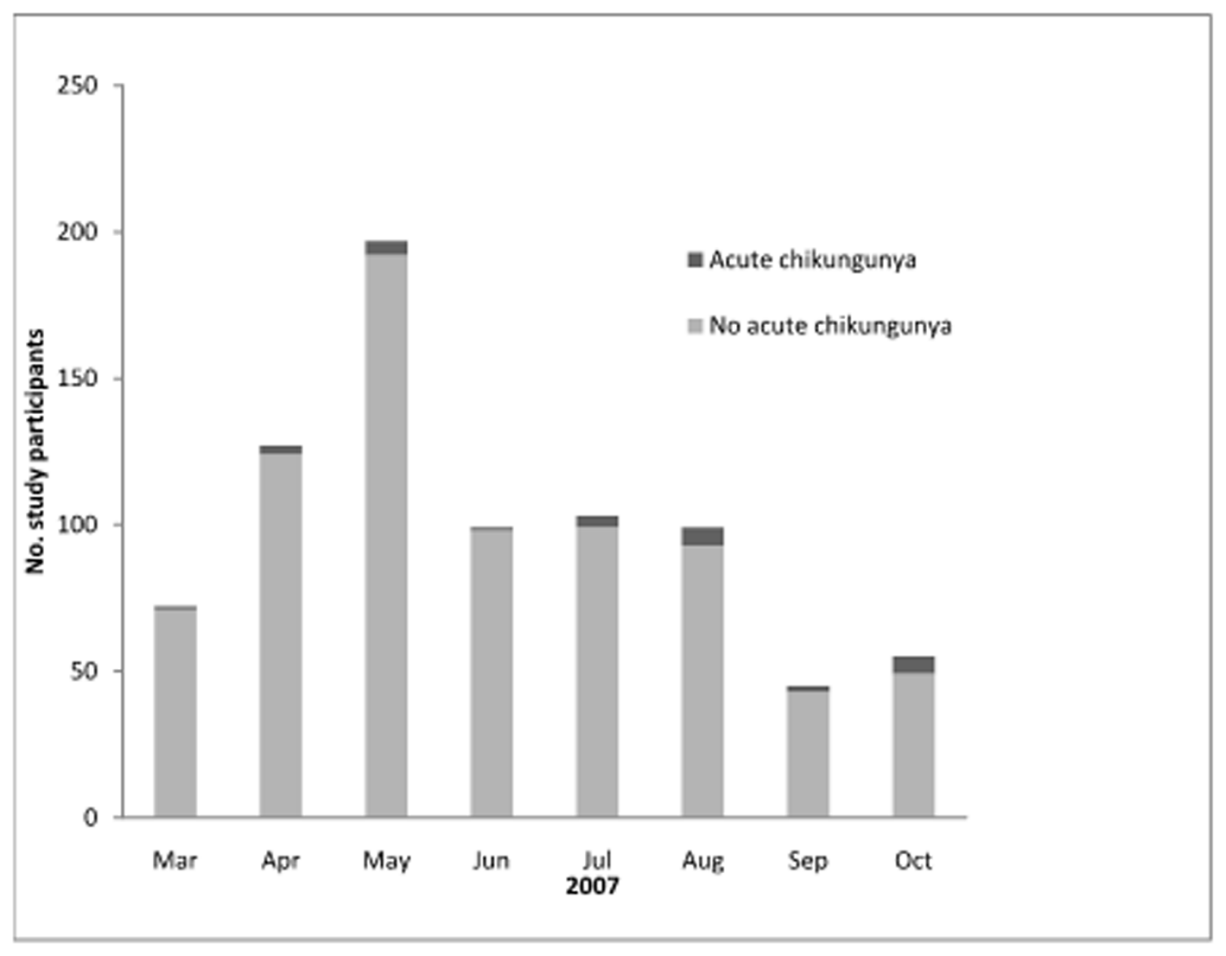
Distribution of febrile patients with and without acute chikungunya infection by month, southern Sri Lanka, 2007.

We identified acute CHIKV infection in 3.5% (28/797) of our febrile cohort, and no CHIKV/DENV co-infections amongst 54 patients with acute DENV described previously [7]. Twenty-six acute CHIKV infections were associated with both a ≥4-fold rise in titer and seroconversion. The age distribution of patients with and without acute CHIKV infection is shown in Figure 1. Cases of acute CHIKV infection occurred during each month of the study, accounting for 1.0% of acute febrile illnesses during June but 10.9% in October (Figure 2). Although there was not a clear monsoon season in 2007 and rainfall was inconsistent (mean 301 mm, range 36 to 657), acute chikungunga infection was more common (p=0.003) in months with more rain (64.3% of cases during typical monsoon months of July to October) than in drier months (35.7% of cases March to June). 

A large proportion of patients with acute CHIKV infection reported taking antibiotics before presentation as did other febrile patients (8 [40%] vs. 201 [35.5%], respectively, p=0.68). Patients with acute CHIKV were more likely (92.9% vs. 70.7%, p=0.01) than those with other febrile illnesses to be admitted to hospital, but the duration of hospitalization tended to be shorter (median 3 days [IQR 2,4] versus 4 days [IQR 3,6], p=0.06). Presumptive clinical diagnosis on presentation was available for 734, including all 28 with acute CHIKV infection. Notably, none of these 28 were initially suspected to have acute CHIKV infection; presumptive diagnoses included non-specific viral syndrome (17), leptospirosis (6), DENV (1), hepatitis (1), infective endocarditis (1), meningitis (1), and gastroenteritis (1). Specified discharge clinical diagnoses (available for 6 patients) included non-specific viral syndrome (2), leptospirosis (1), leptospirosis and CHIKV infection (1), DENV (1), and systemic lupus erythematosus (1). 

### Acute CHIKV infection vs. DENV vs. other acute febrile illness (AFI)

Acute DENV infection in this febrile cohort has been detailed elsewhere (54 patients with DENV confirmed by ELISA, neutralization, isolation, and RT-PCR with acute primary and secondary DENV found to be similar) [7]. Clinical features associated with acute CHIKV infection vs. acute DENV vs. other AFI are shown in Table 1. This 3-way comparison was chosen because comparing acute CHIKV with other AFI including DENV (2-way comparison) yielded similar results, there were no CHIKV/DENV co-infections, and clinical differentiation of the two has been difficult. 

**Table 1:** Clinical characteristics of febrile patients with acute chikungunya vs. acute dengue vs. other acute febrile illness, southern Sri Lanka, 2007.

**Clinical Characteristics**	**Acute chikungunya (n=28)**	**Acute dengue (n=54)**	**Neither infection (n=766)**	P value*
Age in years, median (IQR)	41 (29, 51)	28 (22, 45)	31 (19, 48)	0.14
Adult (≥ 18 years)	96%	89%	79%	0.02
Male	82%	69%	60%	0.04
Days ill, median (IQR)	3.0 (2.0, 3.5)	4.0 (2.0, 5.0)	3.0 (2.0, 7.0)	0.05
Days fever, median (IQR)	3.0 (2.0, 3.0)	4.0 (3.0, 7.0)	3.0 (2.0, 5.0)	0.17
Prior antibiotic treatment	40%	50%	35%	0.16
Admitted to hospital	93%	93%	71%	<0.001
**Symptom**				
Headache	75%	76%	78%	0.87
Sore throat	18%	13%	29%	0.02
Cough	15%	35%	60%	<0.001
Dyspnea	11%	15%	17%	0.64
Lethargy	52%	69%	67%	0.25
Joint pain	71%	51%	43%	0.009
Muscle pain	82%	58%	47%	0.001
Abdominal pain	11%	25%	19%	0.30
Vomiting	32%	43%	37%	0.56
Diarrhea	18%	22%	10%	0.02
**Sign**				
Conjunctival infection	36%	28%	13%	<0.001
Pharyngeal exudate	7%	6%	14%	0.11
Lymphadenopathy	18%	4%	24%	0.002
Jaundice	0%	8%	1%	0.004
Lung crackles	4%	6%	14%	0.058
Tender abdomen	7%	19%	9%	0.08
Hepatomegaly	4%	11%	5%	0.14
Rash	11%†	6%	2%	0.007
Arthritis	0%	2%	0.5%	0.40
**Laboratory parameter**				
WBC, median (IQR)	4500 (3500-6400)	5300 (3600-12000)	8100 (6000-11200)	0.002
ANC, median (IQR)	3150 (2223, 5248)	3600 (2304, 8560)	5549 (3724, 8150)	0.0001
ALC, median (IQR)	1305 (969, 1728)	1602 (1044, 2400)	2109 (1575, 2848)	0.0001
Hemoglobin, mean +/-SD	13.7+/-1.6	12.9+/-1.6	12.6+/-1.7	0.0008
Platelets, median (IQR)	181 (147, 256)	190 (156, 242)	234 (190, 297)	0.0001

WBC, White blood count per μl; ANC, Absolute neutrophil count per μl; ALC, Absolute lymphocyte count per μl; IQR, interquartile range; SD, standard deviation; Hemoglobin, g/dL; Platelets, x1000/μl †1 urticaria, 1 eschar *Kruskal-Wallis test for proportions and skewed continuous variables; analysis of variance test: normally distributed continuous variables.

Patients with acute CHIKV infection and other AFI reported a slightly shorter duration of febrile illness (both 3 days) than did patients with DENV (4 days, p=0.05). Patients with acute CHIKV infection were older (all but one patient ≥18 years of age, p=0.02) and more likely to be male (82% versus 60-69% with DENV or other AFI). Muscle pain and joint pain were common symptoms (82% and 71%, respectively) in those with acute CHIKV, and more frequent than in patients with DENV or other AFI (p<0.001 and p=0.009, respectively). Those with acute CHIKV were as likely to report headache as those with DENV or other AFI (all ≥ 75%), but much less likely to report cough (15% CHIKV vs. 35% DENV vs. 60% other AFI, p<0.001). On examination, conjunctivitis and rash were uncommon features (36% and 11%, respectively), but more often present in those with acute CHIKV vs. those with DENV or other AFI (p=0.001 and p=0.02, respectively). Those with CHIKV had lower white blood cell (including neutrophil and lymphocyte subsets) and platelet counts (p=0.002 and 0.0001, respectively) than those with DENV or other AFI. The unadjusted odds of joint pain and of muscle pain in those with acute CHIKV infection vs. non-dengue AFI were 5.1 (95% CI 1.9-13.6) and 3.3 (95% CI 1.4-7.5), respectively; the unadjusted odds of musculoskeletal symptoms were increased in those with acute CHIKV even relative to acute DENV [joint pain OR 2.4 (95% confidence interval 0.9-6.4) and muscle pain OR 3.4 (95% confidence interval 1.1-10.3)]. Although most patients reported resolution of symptoms at follow-up, patients with acute CHIKV infection were more likely than patients with DENV or other AFI to report persistent muscle and joint pain at the convalescent (range 12- 45 days after acute visit) visit (11% vs. 8% vs. 2%, p=0.003 and 14% vs. 4% vs. 3%, p=0.002, respectively). 

### Past CHIKV infection

**Figure 3:**
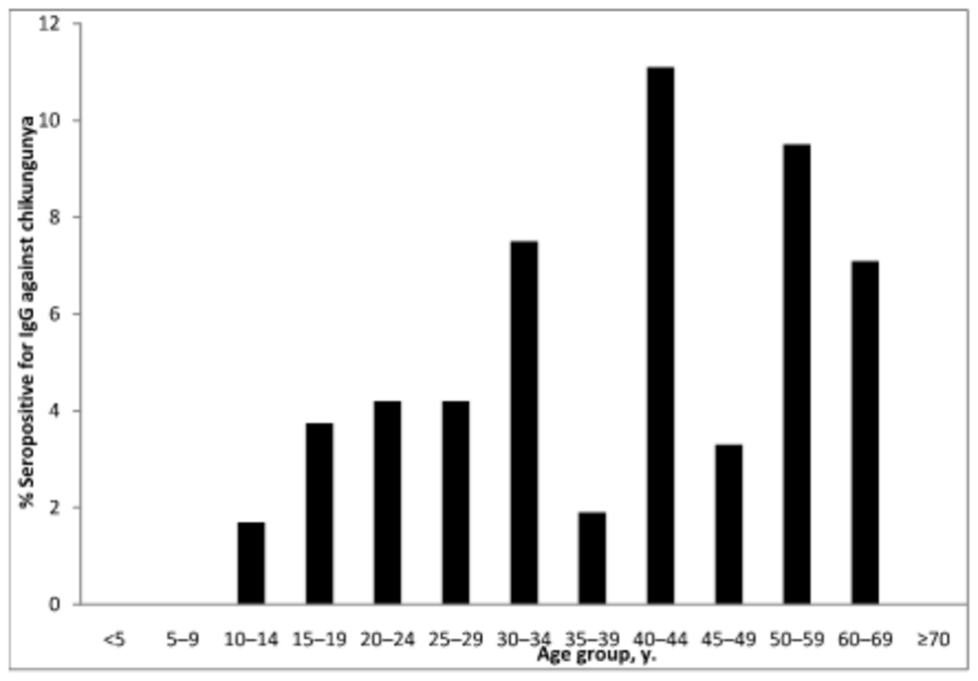
Presence of anti-chikungunya IgG by age (years) in febrile patients, southern Sri Lanka, 2007.

Only 12 (1.5%) patients were found to have serologic evidence of past CHIKV infection. Acute and convalescent titers were relatively low (64 and 64, respectively, in 7 patients, 32 and 64 in 3 patients, 128 and 64 in 1 patient, and 128 and 128 in 1 patient). The age distribution of those with serologic evidence of past CHIKV infection is shown in Figure 3. The demographic characteristics of the 40 patients with acute or past CHIKV infection vs. no evidence of CHIKV infection are detailed in Table 2. Those with evidence of past CHIKV infection were older than those with acute infection, and both were older than those with no evidence of CHIKV infection (median age 49 years [IQR 21-57] vs. 41 [IQR 29-51] vs. 30 [IQR 19-48], respectively, p =0.05). Similarly, the proportion that were male varied significantly among the groups (75% vs. 82% vs. 60%, p=0.03). Although uncommon, urban residence was reported more frequently in those with acute or past CHIKV infection vs. those with no evidence of CHIKV infection(17.5% vs. 7.9%, p=0.03). 

**Table 2:** Demographic characteristics of febrile patients with serologic evidence of acute or past chikungunya infection vs. no serologic evidence of chikungunya, Southern Sri Lanka, 2007*.

**Demographic Characteristics**	**Chikungunya (n=40)**	**No chikungunya (n=757)**	**P value†**
**Age** in years, median (IQR)	41.6 (27.8-51.8)	30.3 (18.5-47.7)	**0.01**
**Male**	80.0	59.7	**0.01**
**Residence**			**0.03**
Rural	82.5	92.1	
Urban	17.5	7.9	
**Type of Work**			**0.01**
Home	23.1	26.9	
Laborer	28.2	25.3	
Farmer, e.g. rice paddy	2.6	2.7	
Merchant	10.3	2.7	
Student	5.1	23.1	
Other	30.8	19.3	
**Swim/Bathe/Wade**			**0.93**
None	76.9	74.2	
River	10.3	13.0	
Paddy Field	10.3	10.2	
Pond/Lake	0	1.1	
Other	2.6	1.6	
**Water Source**			**0.05**
Tap	50.0	29.5	
Boiled	7.5	9.6	
Well	42.5	60.3	
Other	0.0	0.7	

* Except for median age, data are percentages. IQR, intraquartile range

† Kruskal-Wallis test to compare proportions across groups.

### Confirmation of acute CHIKV infection

We isolated virus from 10 of 28 (35.7%) patients with acute CHIKV infection, including 7 of 15 (73.3%) with seroconversion and convalescent titer of 64 versus 1 of 11 (9.1%) with seroconversion and convalescent titer ≥128. Duration of illness and fever before presentation were similar in viremic and non-viremic individuals. Samples were RT-PCR-positive in 18/28 (64.3%) of patients with acute CHIKV infection, including all 10 from whom virus was isolated (p=0.004). Those in whom virus was identified by RT-PCR reported shorter durations of fever before presentation than RT-PCR-negative patients (mean 2.6+/- 1.0 vs. mean 3.7+/-1.6 days, p=0.04). 

### Phylogenetic analysis of Sri Lankan CHIKV isolates

**Figure 4:**
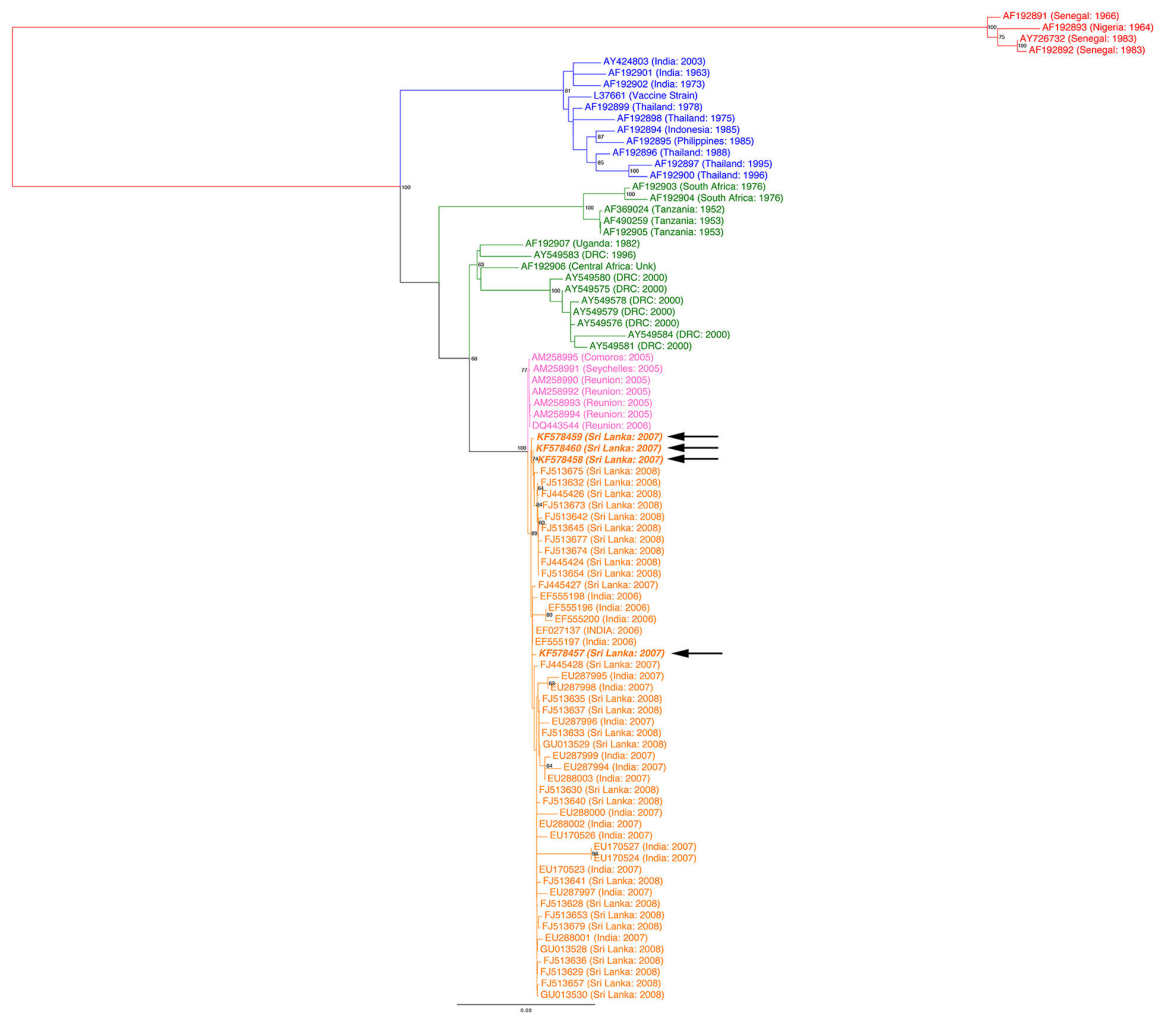
Phylogenetic relationship of Sri Lankan chikungunya virus (CHIKV) isolates. Sequences corresponding to the CHIKV structural genes from the six Sri Lankan isolates were compared to published CHIKV sequences [6,8,10,11,16]. Region of isolation is indicated by text color: West Africa (Red), Asia (Blue), East/South/Central Africa (Green), Reunion Island (Pink) and India/Sri Lanka (Orange). Isolates from the current study are indicated in bold-italics.

The structural coding region of six CHIKV isolates obtained from April to October 2007 (GenBank numbers KF578457-KF578462) were sequenced and compared to other CHIKV sequences deposited in GenBank [6,8,10,11,16]. Phylogenetic analysis shows that the newly sequenced Sri Lankan isolates belonged to the clade that contained viruses isolated from Reunion Islands (2005), Sri Lanka (2007, 2008) and India (2007) (Orange and Pink in Figure 4) with strong bootstrap support (100%) [6,8,10,11,16]. These viruses were distantly related to viruses previously isolated from the Indian subcontinent and SE Asia during 1975–2000 (Blue in Figure 4) and other lineages previously isolated from West Africa during 1975–2000 (Green in Figure 4), highlighting the introduction of a divergent CHIKV lineage to the Indian subcontinent during 2007 [6,8,10,11,16]. Within the new subcontinental lineage, the 2007–2008 Sri Lankan and Indian isolates formed a monophyletic lineage (89% bootstrap support) indicating a common source for these viruses, whereas the 2005 Reunion isolates formed an outlying relationship to the sub-continental strains. These results suggest that the CHIKV that caused the 2007–2008 outbreaks in India and Sri Lanka were most likely derived from Reunion/2005-like isolates.

**Figure 5:**
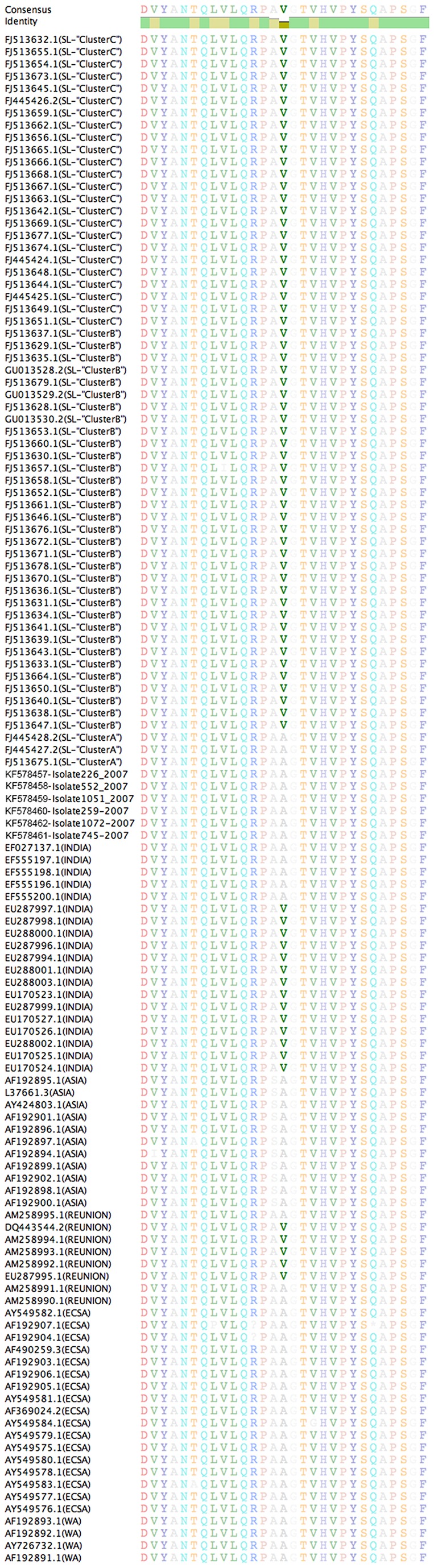
Alignment of the amino acid sequence surrounding the chikungunya virus E1-226 region. Amino acid sequence corresponding to the E1 gene was translated from the nucleotide sequences of the three Sri Lankan isolates and compared the amino acid sequences of the isolates described in Schuffennecker et al 2006, PLoS Med and Kumar et al 2008, JGV [10,11]. Position E1-226 is highlighted (E1-226 corresponds to position 1035 in the polyprotein).

Comparison of amino acid sequence alignments surrounding the E1-226 region for our 6 CHIKV isolates and others from elsewhere in Sri Lanka (Central and Western Province) and India (Figure 5) supports the results obtained through phylogenetic analysis. The isolates we sequenced shared on average 99.69% (standard deviation [SD] 0.22%), 99.71% (SD 0.14%), and 99.65% (SD 0.22%) identity to Sri Lankan clusters A, B, and C, respectively, as described by Hapuarachi et al [6].  These isolates were slightly less similar to the Indian and Reunion Island isolates (99.52% [SD 0.3%] and 99.51% [SD 0.17%], respectively) and lacked the E1-A226V mutation of the latter [6,10]. This E1-A226V was shown to increase the ability of CHIKV to propagate in *Aedes albopictus* and has been implicated in the explosive spread of CHIKV on the Indian Ocean islands and Indian subcontinent [6,10,11,17].

## Discussion

We sought to document whether CHIKV was an important cause of non-specific acute febrile illness in southern Sri Lanka in 2007 and to describe its epidemiological, clinical, and virologic features. We identified the E1-226A strain of CHIKV in southern Sri Lanka in 2007 rather than the E1-A226V strain found in 2008 [6]. This is consistent with other studies from elsewhere in Sri Lanka during the same time period [6]. The CHIKV isolates we sequenced appeared to be most closely related to the Sri Lankan CHIKV clusters A, B and C and those from India isolates described by Hapuarachi et al, and more distantly related to the Reunion Island isolates [6,11]. 

The small (5.0%) proportion of patients with serologic evidence of acute or past CHIKV infection compared with that of DENV (54%) [7] suggests the more recent emergence of CHIKV in southern Sri Lanka, and is consistent with a retrospective serosurvey conducted in Sri Lanka’s Central Province [18]. The finding that most (26/28) acute infections were associated with seroconversion and that all but one patient was ≥18 years old also suggests a population lacking immunity to CHIKV. The finding that males were more likely to have acute CHIKV may reflect more time outdoors; however, it is unclear why more a greater proportion of those with acute CHIKV than acute DENV were male, since both share the same vector.

We describe a milder spectrum of symptomatic CHIKV infection than the epidemic disease reported from sites in the Indian Ocean with mutated virus (E1-A226V). For example, patients in our study had arthralgia but not arthritis, whereas arthritis has been an important feature of E1-A226V-associated epidemic disease [19]. Although milder disease could be explained by a less virulent virus (E1-A226), it may also be related to other factors, such as our enrollment of unselected febrile patients to identify unsuspected CHIKV infections that closely mimicked DENV and other febrile illnesses [7,20]. Severe disease [5] was reported elsewhere in Sri Lanka during the same period that was likely also caused by E1-A226, since E1-A226V appeared in Sri Lanka in 2008 [6]. However, selective recruitment and diagnostic uncertainty (e.g., IgM is less specific than paired IgG) confounds comparison of disease severity in Kularatne’s 2006-2007 cohort versus our Sri Lankan cohort. Our findings emphasize the challenges of clinical diagnosis, particularly in unselected patients with recent onset fever and illness (median 3 days) in the absence of a recognized local epidemic. Clinical acumen is also difficult to hone when confirmatory testing is not available, which highlights the need for affordable, accurate point-of-care diagnostic tests [21]. 

Clinical features suggestive of acute CHIKV infection in our study included almost universal arthralgia and/or myalgia, which was more common and more likely to be persistent even relative to those with acute DENV infection as has been noted by others [16,22,23]. We also found conjunctivitis and rash to be more frequent in those with CHIKV versus DENV [24].. Arthritis was not observed, in contrast to what was found by Kularatne and others as well as reports from the Indian Ocean [5,19,25]. In our febrile cohort of predominantly adults, patients with acute CHIKV infection also presented earlier in illness than patients with DENV, were older, and were disproportionately male. Most studies have included either adults [23] or children [26] but not both. In Tanzania, where children and adults were enrolled in equal proportions, CHIKV was more common in children, which differs from our findings [27]. However, in this study, in which patients were also enrolled with undifferentiated fever, unsuspected acute CHIKV infection and relatively mild illness were also observed [27]. Of note, in Tanzania, acute CHIKV infections occurred more often during dry months and only hepatomegaly and absence of vomiting were associated clinical features. However, other studies with different enrollment criteria (including patients living in tropical countries as well as travelers) have reported acute onset or shorter duration of illness [28], older age [5,23,29], arthritis and/or arthralgia [5,24,28,30], conjunctivitis [31], and rash [24,26,28] to be suggestive of acute CHIKV infection rather than DENV or other acute febrile illness. In contrast, male predominance [5,23,28] and the occurence and degree of leukopenia [32], including lymphopenia, and thrombocytopenia [24,26,30] have been more variable findings. 

Strengths of our study include reproducible and objective enrollment criteria (documented fever of defined magnitude) and use of gold standard diagnostic criteria (paired IgG serology by IFA) made possible by a high proportion (80%) in whom convalescent clinical and serological follow-up was achieved. Convalescent serological follow-up is important, since acute-phase IgM depends critically on the timing of sample collection (insensitive early in acute infection and non-specific during convalescence) and some assays have been found to be unreliable [33]. Our study also highlights the continued role of paired serology, since neither real-time RT-PCR nor direct viral isolation were able to detect all paired sera-confirmed acute CHIKV infections. Paired serology provides only indirect evidence of infection. However, the proportion additionally confirmed by other methods (35.7% by virus isolation and 64.3% by RT-PCR), the association between RT-PCR positivity and shorter duration of illness before presentation, and the improved sensitivity of RT-PCR vs. viral isolation are all consistent with other reports and the known duration (4-7 days) of CHIKV viremia [22,31]. Sequencing to identify circulation of E1-226A CHIKV in southern Sri Lanka in 2007 extends the understanding of the molecular epidemiology of the virus. Finally, we sought but did not find patients co-infected with both CHIKV and DENV, although co-infections could plausibly occur from the bite of a dually-infected *Aedes spp.* mosquito and have been reported [3,34]. 

We were not able to describe the full clinical spectrum of acute CHIKV infection, which would require a large population-based study to capture asymptomatic and minimally symptomatic infections. Since all patients enrolled had fever, we could not have detected “pure arthralgic” (no fever) or “unusual” (neither fever nor arthralgias) forms of CHIKV , which a study in Gabon found in 12.6% and 2.2% cases of confirmed CHIK, respectively [32]. Sample size may have limited our ability to detect a difference in median age and intraquartile range between patients with acute CHIKV infection versus DENV; however, that a greater proportion with acute CHIKV were >18 years suggests that those with CHIKV were older. We did not have the statistical power to contrast clinical features in different age groups, since despite efforts we achieved limited enrollment of children. These limitations notwithstanding, we do describe clinically significant illness in a large cohort in whom selection bias was unlikely (public hospital to which all social strata have access). Further, asymptomatic acute infections are relatively rare (3-25%) with CHIKV compared with DENV [1]. Our estimate of acute and past infections may be conservative. Confirmation of cases by viral isolation and RT-PCR make it unlikely that the cases described were due to cross-reacting alphaviruses. However, we cannot exclude the co-circulation of other strains of CHIKV, since we did not perform sequencing on all isolates. In screening convalescent sera at a 1:32 dilution for anti-CHIKV antibody before testing paired sera, we may have missed a small number of patients with low-titer infections. Additionally, rescreening or testing sera at a lower dilution may have identified a few additional low-titer positives. It is unlikely that we missed past infections (low-titer antibody in the acute sample but not the convalescent sample), since it is unlikely that genuine antibody would be lost in 2-4 weeks. 

In conclusion, we describe the epidemiology and clinical features of acute CHIKV infection associated with wild-type (E1-226A) CHIKV compared with acute DENV and other acute febrile illness in a prospective cohort of febrile patients in southern Sri Lanka. Greater awareness of acute CHIKV infection and the availability of reliable and affordable diagnostic tests to diagnose acute infection are needed in Sri Lanka and elsewhere. Countries in other regions are also at risk [24,35], since outbreaks of DENV, also transmitted by *Aedes* mosquitos, have occurred in both the United States and Europe in the past decade, and one outbreak of acute chikingunya infection in a temperate region has already occurred [31]. 

## Supporting Information

Table S1 **Primer sequences used to amplify and sequence the Sri Lankan chikungunya virus isolates.**
(DOCX)
