# Supplementary material for: Chikungunya as a Cause of Acute Febrile Illness in Southern Sri Lanka
Source: PLoS One. 2013 Dec 2;8(12):e82259. doi: 10.1371/journal.pone.0082259 (PMC3846738; doi:10.1371/journal.pone.0082259)
Supplement: Table S1 — Primer sequences used to amplify and sequence the Sri Lankan chikungunya virus isolates. (DOCX) [file pone.0082259.s001.docx]

**Supporting Information**

**Table S1**. Primer sequences used to amplify and sequence the Sri Lankan chikungunya virus isolates.

| **Name** | **Sequence** | **Position** |
| --- | --- | --- |
| CHIKV-34F | GAGAAGCTCAGAGGACCCGTC | 7455-7475 |
| CHIKV-39R | TGGCACGARTGCCCTTCTCC | 8609-8628 |
| CHIKV-36F | GAGAGRATGTGCATGAAAAT | 7874-7893 |
| CHIKV-41R | TTGCAGTTATTRATCATCACTTTRTC | 9204-9182 |
| CHIKV-40F | GAGGAGATAGAGGTRCAYATG | 9035-9055 |
| CHIKV-45R | CCTTGGTAAAGGACGCGGAG | 10391-10410 |
| CHIKV-44F | ATGTGGGGCGGCGCCTACTG | 10256-10275 |
| CHIKV-49R | GTGYCCCCTAAGAGACACA | 11471-11489 |
